# The emerging role of GATA transcription factors in development and disease

**DOI:** 10.1017/erm.2016.2

**Published:** 2016-03-08

**Authors:** Marjolein HFM Lentjes, Hanneke EC Niessen, Yoshimitsu Akiyama, Adriaan P de Bruïne, Veerle Melotte, Manon van Engeland

**Affiliations:** 1 Department of Pathology, GROW – School for Oncology and Developmental Biology, Maastricht University Medical Center, Maastricht, The Netherlands; 2 Department of Molecular Oncology, Graduate School of Medicine and Dentistry, Tokyo Medical and Dental University, Tokyo, Japan

## Abstract

The GATA family of transcription factors consists of six proteins (GATA1-6) which are involved in a variety of physiological and pathological processes. GATA1/2/3 are required for differentiation of mesoderm and ectoderm-derived tissues, including the haematopoietic and central nervous system. GATA4/5/6 are implicated in development and differentiation of endoderm- and mesoderm-derived tissues such as induction of differentiation of embryonic stem cells, cardiovascular embryogenesis and guidance of epithelial cell differentiation in the adult.

The importance of GATA factors for development is illustrated by the embryonic lethality of most single *GATA* knockout mice. Moreover, *GATA* gene mutations have been described in relation to several human diseases, such as hypoparathyroidism, sensorineural deafness and renal insufficiency (HDR) syndrome, congenital heart diseases (CHDs) and cancer. GATA family members are emerging as potential biomarkers, for instance for the risk prediction of developing acute megalokaryblastic leakemia in Down syndrome and for the detection of colorectal- and breast cancer.

## The origin and molecular structure of the GATA family

**Figure 1.**
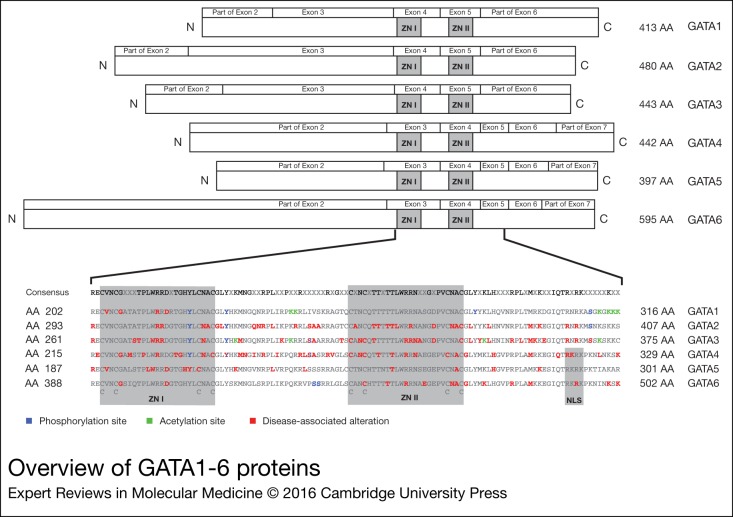
Overview of GATA1-6 proteins. The GATA proteins are depicted in the upper part of the figure. The GATA proteins are aligned according to the location of the zinc fingers (ZNI and ZNII). The exon boundaries are depicted above the protein structure. For GATA4 the TADI and TADII are shown. In the lower part of the figure the regions around the zinc fingers are enlarged, with the correspondingAA numbers written next to the GATA sequence. Posttranslational modification (post-transciptional modification) sites and disease-associated alterations are marked on top of the corresponding AA. AA, amino acid; TAD, transcriptional activation domains.

In vertebrates, six GATA transcription factors have been identified. Based on phylogenetic analysis and tissue expression profiles, the GATA family can be divided into two subfamilies, GATA1/2/3 and GATA4/5/6 (Ref. 1). Although in non-vertebrates *GATA* genes are linked together onto chromosomes, in humans they are segregated onto six distinct chromosomal regions (Table 1), indicating segregation during evolution (Ref. 2). Most *GATA* genes encode for several transcripts and protein isoforms. GATA proteins have two zinc finger DNA binding domains, Cys-X_2_-C-X_17_-Cys-X_2_-Cys (ZNI and ZNII), which recognise the sequences (A/T)GATA(A/G) (Fig. 1) (Ref. 3). Amongst the six GATA binding proteins, the zinc finger domains are more than 70% conserved, while the sequences of the amino-terminal and carboxyl-terminal domains exhibit lower similarity (Ref. 4). In non-vertebrates GATA transcription factors have been identified that contain mostly one zinc finger, i.e. in *Drosophila melanogaster* and *Caenorhabditis elegans* (Ref. 3). The C-terminal zinc finger (ZNII) exists in both vertebrates and non-vertebrates indicating that ZNI was duplicated from ZNII (Ref. 2).

**Table 1.** Molecular features of the human GATA transcription factors

	Genomic sequence			mRNA sequence			Protein sequence		
Name	Chromosomal location	Ensembl acession no.	CpG island in the promoter region	Transcripts	Ensembl transcript ID^a^	Coding exons	Uniprot accession no.	Isoform	Protein length (AA)
GATA1	Xp11.23	ENSG00000102145	None	3	ENST00000376670	5	P15976	1	413
								2	330
								3	335
GATA2	3q21.3	ENSG00000179348	+	6	ENST00000341105	5	P23769	1	480
								2	466
GATA3	10p15	ENSG00000107485	+	5	ENST00000346208	5	P23771	1	443
								2	444
GATA4	8p23.1-p22	ENSG00000136574	++	9	ENST00000335135	6	P43694		442
GATA5	20q13.33	ENSG00000130700	++	4	ENST00000252997	6	Q9BWX5		397
GATA6	18q11.1-q11.2	ENSG00000141448	++	1	ENST00000269216	6	Q92908	1	595
								2	449

a In the case of multiple transcripts the ensembl transcript ID was chosen, based on the first isoform of the corresponding Uniprot protein sequence.

## Tissue-specific roles of GATA factors in development and disease

### Haematopoietic system

*GATA1/2/3* knockout mice die at the embryonic stage due to haematological abnormalities (Table 2), indicating a pivotal role of these transcription factors in haematopoietic development (Ref. 1).

**Table 2.** Phenotype of GATA knockout mice

Name	Phenotype (embryonic day)	Abnormality	Reference
GATA1	die (11.5–12.5 dpc)	Defective erythroid cell maturation	(Ref. 5)
GATA2	die (12.5 dpc)	Severe anaemia	(Ref. 6)
GATA3	die (11–12 dpc)	Massive internal bleeding and severe deformities of the brain and spinal cord	(Ref. 7)
GATA4	die (9.5 dpc)	Defects of heart morphogenesis and ventral closure of the forgut	(Ref. 8)
GATA5	Viable and fertile	Females exhibited pronounced genitourinary abnormalities that included vaginal and uterine defects and hypospadias	(Ref. 9)
GATA6	die (5.5–7.5 dpc)	defects of visceral endoderm function and subsequent extra-embryonic endoderm	(Ref. 10)

*Dpc*, days post coïtum.

**Figure 2.**
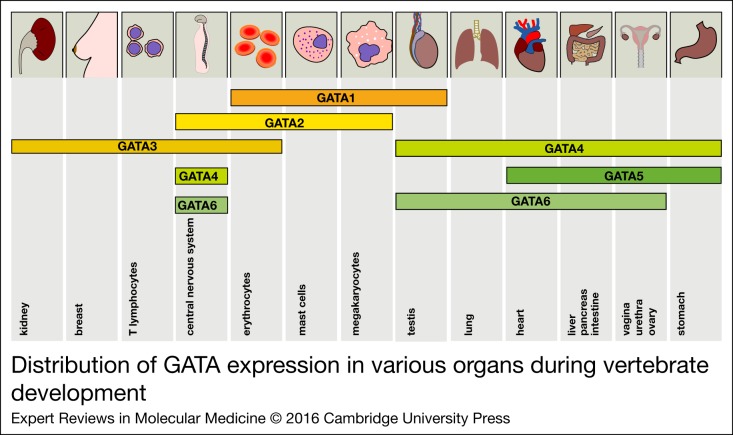
Distribution of GATA expression in various organs during vertebrate development. The expression of all GATA factors is depicted in the corresponding tissues. The distribution of the expression patterns roughly reflects the two GATA subgroups (GATA1/2/3 versus GATA4/5/6).

GATA1, the first recognised member of the GATA family, is specifically expressed during haematopoietic development of erythroid, and megakaryocytic cell lineages (Fig. 2) (Ref. 11). Loss of *GATA1* in mouse embryo-derived stem cells results in a complete lack of primitive erythroid precursor production (Ref. 5). Definitive erythroid precursors, on the other hand, are normally produced, but undergo a maturation arrest at the proerythroblast stage followed by apoptosis (Ref. 12). Ablation of *GATA1* in adult mice also results in a maturation arrest at the same proerythroblast stage (Ref. 13). The requirement of the different GATA1 functional domains during primitive and definitive erythropoiesis has been investigated in vivo, showing that both zinc fingers are needed to rescue *GATA1* germline mutant mice (Ref. 14). In haematopoietic stem cells (HSCs), *GATA1* gene expression is suppressed, which is indispensable for the maintenance of these stem cells. The mechanism behind this suppression is not fully understood yet. Recently, it was shown that decreased DNA methylation of the *GATA1* locus leads to increased GATA2 binding and that increased GATA2 binding results in *GATA1* gene transactivation. According to these study results, Takai et al. proposed a mechanism in which *GATA1* hypomethylation results in an accessible locus for GATA2 binding which enables transactivation of *GATA1* gene expression to initiate erythropoiesis in megakaryo-erythroid progenitors (Ref. 15). Loss of *GATA1* results in a marked increase of GATA2 expression, indicating not only that GATA2 partially compensates for GATA1 but also that GATA1 suppresses GATA2 transcription during normal erythropoiesis (Ref. 16). This suppression is mediated by the displacement of GATA2 from its upstream enhancer by increasing levels of GATA1 referred to as the ‘GATA switch’ (Ref. 17). The combined loss of *GATA1* and *GATA2* in double-knockout embryos leads to an almost complete absence of primitive erythroid cells, suggesting functional overlap between these transcription factors early in the primitive erythropoiesis (Ref. 18).

Requirement of functional GATA1 for haematopoiesis is also observed in several human diseases, such as anaemia, leukaemia and thrombocytopenia (Table 3). Splice site mutations of *GATA1* have been found in a family with macrocytic anaemia and in patients with Diamond-Blackfan anaemia (an anaemia characterised by a selective hypoplasia of erythroid cells), resulting in impaired production of the full-length form of the GATA1 protein (Refs 19, 20).

**Table 3.** GATA transcription factors in disease

	Disease	Abberation	Location	Consequence
*GATA1*	XLT	MS mut	ZnF1	FOG1 interaction ↓
	XLTT	MS mut	ZnF1	DNA binding ↓
	Anaemia (e.g. Diamond-Blackfan anaemia)	Splice site mutation, mutation initiation codon	exon 2	Only short for or loss of the full length GATA1 isoform
	Congenital erythropoietic porphyria	MS mut	ZnF1	Unknown
	TMD and AMKL in DS	FS INS and DEL, NS mut and splice site mutation	Intron 1, exon 2 and 3	Protein truncation, transcriptional activation ↓
	AMKL without DS	INS	exon 2	Protein truncation
*GATA2*	Chronic myeloid leukaemia	MS mut, FS DEL	ZnF2	DNA binding ↑, transcriptional activation ↓
	DCML / MonoMAC / Emberger syndrome	FS INS and DEL, MS and NS mut, full gene DEL	ZnF2, 5′UTR, intron 5	Nonfunctional protein, nonsense-mediated decay
	Myelodysplastic syndrome	FS INS and DEL, MS and NS mut, full gene DEL	exon, intron, 5′UTR	Protein truncation, DNA binding ↓
	Acute myeloid leukaemia	MS mut, FS INS, full gene DEL	ZnF1, ZnF2, exons	Nonfunctional protein
*GATA3*	HDR syndrome	MS and NS mut, FS INS and DEL, splice site mutation,partial and full gene DEL	ZnF1, ZnF2, exons	Protein truncation, FOG2 interaction ↓, DNA binding/affinity ↓
	Breast cancer	MS and NS mut, FS INS and DEL	ZnF2, exons	Protein truncation, nonfunctional protein
	T-ALL	MS mut, FS DEL, in-frame DEL	ZnF1, ZnF2	Likely loss of function
	B-ALL	SNP	Intron 3	Unknown
	UCC and RCC	CpG methylation	Promoter	Transcriptional activation ↓
*GATA4*	CHD	MS and NS mut, FS INS and DEL, SNP, full gene DEL, gene duplication	ZnF1, ZnF2, exons, 3'-UTR, introns, promoter	Protein truncation, DNA binding/affinity ↓, transcriptional activation ↓, TBX5 interaction ↓, changed RNA folding
	Pancreatic agenesis	MS mut, intragenic and full gene deletion	ZnF2	Transcriptional activation ↓, DNA binding ↓
	GI cancer	CpG methylation, amplification	Promoter, 8p	Transcriptional activation ↓/↑
	Glioblastoma multiforme	CpG methylation, FS INS and DEL	Promoter, ZnF domains, C terminal region	Transcriptional activation ↓
	Ovarian cancer	Hypoacetylation, loss trimethylation, CpG methylation	Histone 3 and 4, lysine 4	Transcriptional activation ↓
	Other cancers (e.g. lung, DLBCL)	CpG methylation	Promoter	Transcriptional activation ↓
*GATA5*	CHD	MS and NS mut	ZnF1, ZnF2	Transcriptional activation ↓
	Cancer (e.g. GI cancer, RCC)	CpG methylation	Promoter	Transcriptional activation ↓
*GATA6*	CHD	MS and NS mut, duplication and DEL	ZnF1, ZnF2, exons	Transcriptional activation ↓
	Pancreatic agenesis	MS and NS mut, FS INS and DEL	Znf2, exons	Transcriptional activation ↓
	Ovarian cancer	Hypoacetylation, loss trimethylation, upregulation	Histone 3 and 4, lysine 4	Transcriptional activation ↓/↑
	GI cancer	Amplification, CpG methylation	18q, promoter	Transcriptional activation ↓/↑
	Pancreatobiliary cancer	Amplification	18q11.2	Transcriptional activation ↑
	Pediatric rhabdomyosarcoma	CpG methylation	Promoter	Transcriptional activation ↓

AMK, acute megakaryoblastic leukaemia; B-ALL, B-cell acute lymphoblastic leukaemia; CHD, congenital heart disease; CML, chronic myeloid leukaemia; DCML, dendritic cell, monocyte, B-lymphocyte and natural killer lymphocyte deficiency; DEL, deletion; DLBCL, diffuse large B-cell lymphoma; DS, Down syndrome; GI, cancer gastrointestinal cance; FS, frameshift; HDR, hypoparathyreoidism, sensorineural deafness and renal disease; INS, insertion; MS, mut missense mutation; MonoMAC, syndrome associated with monocytopenia, B and NK, cell lymphopenia and mycobacterial, fungal and viral infections; NS, mut nonsense mutation; RCC, renal cell carcinoma; SNP, single nucleotide polymorphism; T-ALL, T-cell acute lymphoblastic leukaemia; TMD, transient myeoloproliferative disorder; UCC, urothelial cell carcinoma; XLT, X-linked thrombocytopenia; XLTT, X-linked thrombocytopenia with thalassemia.

Conditional megakaryocytic lineage specific *GATA1* knockout mice show excessive marrow megakaryocyte proliferation whereas the platelet numbers are decreased. The maturation of these hyperproliferated megakaryocytes is severely impaired and the produced platelets are structurally and functionally abnormal (Ref. 21). Additionally, megakaryocyte-expressed genes with functional GATA1-binding sites (e.g. STAT1) are downregulated in *GATA1^−/−^* megakaryocytes (Ref. 22). Loss of GATA1 leads to overexpression of GATA2 in megakaryocytes. However GATA1-deficient megakaryocytes still show abnormal megakaryocytic proliferation and differentiation, establishing no functional redundancy of these transcription factors in megakaryopoiesis (Ref. 23). In contrast to erythropoiesis, GATA2 remains to be expressed after the GATA switch in late megakaryopoiesis, suggesting a divergent function for both GATA proteins (Ref. 24).

Children with trisomy 21 are at risk of developing leukaemia, in particular acute megakaryoblastic leukaemia (AMKL). Nearly all Down syndrome patients with AMKL harbour somatic mutations in the *GATA1* gene (Table 3) (Ref. 25), predominantly leading to an N-terminal truncated ‘short’ GATA1 protein (GATA1s) (Ref. 26). Inadequate GATA1 mediated repression of specific oncogenic factors contributes to megakaryocytic abnormalities (Ref. 27). Analysis of Down syndrome children with transient myeloproliferative disorder (TMD), which is considered a potential precursor to AMKL, also revealed *GATA1* mutations (Ref. 28). Noticeable the *GATA1* mutation in TMD and subsequent AMKL is identical, suggesting that *GATA1* mutations are early events in the development of AMKL in trisomy 21-children (Ref. 29). Not all TMD Down syndrome neonates with a *GATA1* mutation progress to AMKL, indicating the need for more molecular events contributing to the pathogenesis of AMKL. Recently, Yoshida et al. reported newly acquired driver mutations, which lead to the development from TMD to Down syndrome-AMKL (Refs 30, 31).

The mechanism behind the leukaemogenesis remains elusive. Based on mutational spectrum analysis of the *GATA1* locus in Down syndrome AMKL, Cabelof et al. hypothesised that increased oxidative stress because of trisomy 21, uracil accumulation and reduced DNA repair together driving leukaemogenesis in Down syndrome (Ref. 32). Recently it was shown that *GATA1* mutations protect megakaryocytes from activated AKT-induced apoptosis (Ref. 33). Additionally, trisomy 21 itself increases HSC frequency, clonogenicity and megakaryocyte-erythroid output with associated megakaryocyte-erythroid progenitor expansion (Refs 34, 35, 36). Another hypothesis is that upregulation of runt-related transcription factor 1 (RUNX1), which physically interacts with GATA1, due to trisomy 21 leads to the induction of GATA1 transcription during embryogenesis, thereby leading to transcription-associated mutagenesis (Ref. 37). Recently it is shown that loss of type I interferon (IFN) signalling contributes to GATA1s-induced megakaryocyte hyperproliferation, suggesting AMKL-treatment with IFN-α administration (Ref. 38).

*GATA1* mutations are also detected in a specific form of X-linked hereditary thrombocytopenia and are described with and without thalassemia (Table 3 and Supplemental Table 1). Hereditary thrombocytopenia without thalassemia has been associated with *GATA1* missense mutations that are located in the N-terminal zinc finger region. These mutations lead to loss or inhibition of GATA1 interaction with friend-of-GATA(FOG)1-cofactor (Ref. 39). The degree of disrupted GATA1–FOG1 interaction depends on the mutation, explaining different clinical presentations (Ref. 40). The only *GATA1* mutation reported in hereditary X-linked thrombocytopenia with thalassemia is the missense mutation R216Q which is located in the DNA binding surface of the GATA1 N-terminal zinc finger and results in reduced DNA binding rather than affecting GATA1–FOG1 interaction (Ref. 41).

In vertebrates, *GATA2* is expressed in haematopoietic progenitor cells (HPCs), early erythroid cells, mast cells and megakaryocytes, closely resembling the cellular distribution of GATA1 (Fig. 2). A deficit in primitive erythropoiesis is apparent in *GATA2^−/−^* mice since the total number of blood cells during embryonic development is markedly reduced, leading to lethality because of severe anaemia (Table 2) (Ref. 6). In *GATA2^+/−^* mice haematopoietic defects are seen within HSCs and granulocyte-macrophage progenitor cells. Moreover, the loss of *GATA2* in adult mice leads to profound abnormalities in definitive haematopoiesis, also directing to a defect at the level of HSCs (Refs 6, 42, 43). The function of GATA2 in haematopoietic development has recently been reviewed by Bresnick et al. (Ref. 44), describing GATA2 as one of the key components establishing the transcriptional program for early haematopoietic development.

Two different *GATA2* alterations have been reported in patients with chronic myeloid leukemia (CML) during blast crisis formation (Table 3). In contrast to the in-frame deletion Δ341-346, which leads to decreased transcriptional activation, *GATA2* L359V is a gain-of-function mutation and leads to increased DNA binding. Transduction of *GATA2* L359V (in vitro and in vivo) resulted in disturbed myelomonocytic differentiation/proliferation, suggesting *GATA2* mutations are involved in the acute myeloid transformation of CML (Ref. 45).

*GATA2* gene mutations that predisposed to myelodysplastic syndrome (MDS) and acute myeloid leukaemia (AML) were reported (Supplemental Table 1). This occurred either in the absence (non-syndromic) or presence of certain syndromes, including Emberger syndrome and monoMAC syndrome (Ref. 46). Most mutations affect the C-terminal zinc finger or result in N-terminal frameshift mutations (Ref. 47).

Similar expression patterns of GATA1, GATA2 and GATA3 in human, murine and avian erythroid cells indicate a conserved role for these GATA transcription factors in vertebrate erythropoiesis (Ref. 48). Beyond its expression in erythroid lineages, GATA3 is also expressed in T lymphocytes (Ref. 49). During haematopoiesis vertebrate GATA3 is expressed in HSCs and in developing T lymphocytes. Murine *GATA3^−/−^* embryos are predominantly affected during definitive haematopoiesis in the fetal liver. Although later than *GATA2^−/−^* mice, these embryos appear also anaemic and die in utero, probably owing to massive internal bleeding (Table 2) (Ref. 7). Frelin et al. demonstrated that GATA3 regulates the self-renewal and differentiation of bone marrow long-term HSCs (Ref. 50). During embryogenesis, GATA3 deficiency leads to a marked reduction in the production of HSCs in the aorta-gonads-mesonephros region. It was shown that GATA3 regulates HSC emergence during embryogenenis via the production of catecholamines linking the haematopoietic system development to the development of the sympathetic nervous system (SNS) (Ref. 51).

In T cell development, GATA3 has a pivotal role from the generation of early T lineage progenitors to CD4^+^ specification [as reviewed in (Ref. 52)]. During antigen presentation by specialised antigen-presenting cells, the TCR is stimulated, thereby driving differentiation from peripheral naïve CD4^+^ T cells towards T helper cell type 1(T_H_1) or 2 (T_H_2). GATA3 expression in differentiating T_H_2 cells is mediated by different pathways as clearly reviewed in Ho et al. (Ref. 53). GATA3 and STAT6 in T_H_2 lineage account for lineage specific expression of T cell lincRNAs. At the moment, the function of lincRNAs during T cell development and differentiation is under investigation (Ref. 54). An essential function for GATA3 beyond T_H_2 differentiation is also described demonstrating GATA3 controls proliferation and maintenance of mature T cells (Ref. 55).

GATA3 dysregulation is described in leukaemia. Together with T-cell acute lymphocytic leukemia 1 (TAL1) and RUNX1, GATA3 forms an autoregulatory loop that positively regulates the v-myb avian myeloblastosis viral oncogene (MYB) oncogene, which in turn controls the gene expression program in T-cell acute lymphoblastic leukaemia (T-ALL) (Ref. 56). Thereby, whole-genome sequencing of patients with early T-cell precursor ALL, an aggressive subtype of T-ALL, revealed *GATA3* inactivating mutations (Supplemental Table 1) (Ref. 57).

In summary, GATA1/2/3 are essential regulators in the development of erythroid and megakaryocytic cell lineages and in the molecular pathogenesis of different haematopoietic diseases.

### Cardiovascular system

The mesoderm gives rise to numerous organs, including the heart and genitourinary tract. GATA4/5/6 proteins are expressed in the mesodermal precursors that develop into the heart (Ref. 58).

GATA4 is one of the earliest transcription factors expressed in developing cardiac cells, already detectable in murine precardiac splanchnic mesoderm and associated endoderm (Ref. 8). *GATA4^−/−^* mice display severe defects in ventral foregut closure and heart morphogenesis, resulting in embryonic lethality at embryonic day 8 (Table 2). These deformities result from a general loss in ventral folding throughout the embryo and implicate GATA4 requirement for the migration or folding morphogenesis of the precardiogenic splanchic mesodermal cells (Ref. 8). Mice harbouring a knock-in mutation that abrogates the interaction with FOG-cofactors *(GATA4^Ki/Ki^)* lack coronary vessels (Ref. 59). In addition, murine GATA4 regulates cardiac angiogenesis by inducing angiogenic factors such as VEGF, facilitating compensation following injury (Ref. 60). Yamak et al. have suggested that GATA4 and Cyclin D2 are part of a forward reinforcing loop in which Cyclin D2 feeds back to enhance cardiogenic activity of GATA4 through direct interaction. *GATA4* mutations that abrogate Cyclin D2 interactions are associated with human CHD (Ref. 61).

A variety of *GATA4* mutations have been detected in patients with various forms of CHD such as Tetralogy of Fallot, ventricular septal defect and atrial fibrillation as reviewed by McCulley et al and summarised in Table 3 and Supplemental Table 1 (Ref. 62).

Within the developing heart, GATA5 is expressed in the myocardium as well as in the endocardium and derived endocardial cushions in mouse embryos (Ref. 63). Depending on how *GATA5* is inactivated in several mouse models, different cardiac phenotypes are described. Deletion of both GATA5 isoforms leads to hypoplastic hearts and partially penetrant bicuspid aortic valve formation (Ref. 64). When a *GATA5* mutant allele was established that lacked the two zinc finger domains, cardiovascular defects were only detectable in a *GATA4^+/−^* background (Ref. 65). Although little is known about GATA5 in human heart conditions, three heterozygous *GATA5* mutations have been associated with familial atrial fibrillation (Ref. 66) and four heterozygous *GATA5* mutations with CHD (Ref. 67).

GATA6 is abundantly expressed in vascular smooth muscle cells during murine embryonic and postnatal development (Ref. 68). *GATA6*^−/−^ mice die at the embryonic stage due to defects of the extra-embryonic endoderm (Table 2) (Ref. 10). Tissue-specific deletion of *GATA6* in neural crest-derived smooth muscle cells results in an interrupted aortic arch and persistent truncus arteriosus (PTA). These results suggest that GATA6 is required for proper patterning of the aortic arch arteries. This phenotype is associated with severely attenuated expression of semaphorin 3C, a signalling molecule critical for both neuronal and vascular patterning (Ref. 69). Other GATA6 target genes, e.g. *Wnt2*, in vascular smooth muscle cells and cardiac cells have been identified by microarray analysis after transient GATA6 over-expression. Interestingly, *GATA6* is also a target of Wnt2 and together they form a feedforward transcriptional loop to regulate posterior cardiac development (Ref. 70).

A number of mutations have been described for *GATA6* in the aetiology of CHD (Table 3; Supplemental Table 1). For example, two *GATA6* mutations were found in patients with PTA disrupting the transcriptional activity of the GATA6 protein on downstream genes involved in the development of the cardiac outflow tract (Ref. 71).

Thus, the GATA4/5/6 transcription factors have closely related functions during cardiovascular development, and defects lead to CHD and other heart conditions.

### Gastrointestinal tract

The endoderm gives rise to the respiratory and gastrointestinal tract as well as the associated organs such as pancreas and liver. Differentiation of embryonic stem cells towards the extra-embryonic endoderm can be induced by forced expression of either GATA4 or GATA6 (Ref. 72). Targeted mutagenesis of *GATA4* in mouse embryonic stem cells results in disturbed differentiation of the visceral endoderm, suggesting that GATA4 has a role in yolk sac formation (Ref. 73).

Murine GATA4 is expressed in the proximal but not in the distal small intestine and has an important role in the maintenance of jejunal-ileal identities (Ref. 74). Furthermore, GATA4 is essential for jejunal functions such as fat and cholesterol absorption (Ref. 75). Beuling et al. found that reduction of GATA4 activity in the intestine induces bile acid absorption in the proximal ileum, which can restore bile acid homeostasis in mice with an ileocaecal resection (Ref. 76).

Whereas GATA4 expression is absent from the distal ileum, *GATA6* is expressed throughout the entire small intestine. Conditional deletion of *GATA6* in the ileum results in a decrease of crypt cell proliferation and numbers of enteroendocrine and Paneth cells, an increase in numbers of goblet-like cells in crypts and altered expression of genes specific to absorptive enterocytes. GATA4/6 factors are therefore required for proliferation, differentiation and gene expression in the small intestine (Ref. 77).

In humans, GATA4 and GATA5 are expressed in normal gastric and colon mucosa (Refs 78, 79). In gastric and colorectal cancer (CRC) these genes are frequently transcriptionally silenced by methylation (Refs 80, 78). In addition, we reported that GATA4 and GATA5 exhibit tumour suppressive properties in human CRC cells in vitro (Ref. 80). The potential biomarker capacities of *GATA4* are discussed below.

### Liver and pancreas

In the mouse, the ventral foregut endoderm differentiates to form the parenchymal components of the liver and ventral pancreas. Although *GATA4* has an essential function in embryonic liver development, the protein seems to be dispensable in the adult liver function (Refs 81, 82). *GATA6^−/−^* murine embryos have defects in endoderm differentiation, and show severely attenuated GATA4 expression levels and complete absence of hepatocyte nuclear factor 4 (HNF4) expression in the visceral endoderm, parietal endoderm and liver bud (Ref. 83). HNF4 is a key regulator for complete differentiation of visceral endoderm, hepatocyte differentiation and the epithelial transformation of the liver (Ref. 84). Tetraploid rescue experiments with *GATA6* null mice show that GATA6 is a key regulator for liver bud growth and commitment of the endoderm to a hepatic cell fate (Ref. 83).

Development of the ventral pancreas was, in contrast to the dorsal pancreas, impaired in *GATA4^−/−^* murine embryos using tetraploid rescue experiments. *GATA6^−/−^* embryos show a similar phenotype, although not as severe as that observed in *GATA4^−/−^* embryos (Ref. 81). In humans, the role of *GATA6* in pancreatic development became apparent in a group of patients with pancreatic agenesis, in which Allen et al. identified 15 de novo heterozygous inactivating mutations in *GATA6* (Supplemental Table 1). In addition, these patients suffered from CHD, biliary tract abnormalities, gut developmental disorders, neurocognitive abnormalities and other endocrine abnormalities (Ref. 85). In contrast to these results, Martinelli et al. described that GATA6 is dispendable for pancreas development. However, GATA6 is essential for acinar differentiation and maintenance of adult exocrine homeostasis in mice (Ref. 86). An explanation for this contradiction might be the timepoint of *GATA6* inactivation which is earlier in agenesis patients compared with the mouse model used by Martinelli et al. Together these data show the need for further research to unravel the role of GATA6 in pancreatic development.

In pancreatic cancer, GATA6 is often overexpressed, which correlates with *GATA6* amplification (Table 3) (Ref. 87). Retained GATA6 expression has been shown in gastric, colorectal, esophageal, ovarian and pulmonary cancer cell lines (Refs 78, 88, 89, 90). Additionally, intestinal GATA6 expression is higher in proliferating progenitor cells compared with differentiated cells (Ref. 91). In primary gastric cancer, the pro-oncogenic effects of GATA6 are recently confirmed, in vitro and in vivo (Ref. 92).

### Urogenital tract and kidney

GATA1 is abundantly expressed in the Sertoli cells of the testis during murine prepubertal testis development (Fig. 2). GATA1 expression decreases thereafter and is in the adult mouse testis only found in the Sertoli cells during different stages of the spermatogenesis (Ref. 93). Surprisingly, Sertoli-specific *GATA1* knockout mice show no alterations in testis development, spermatogenesis, male fertility and expression of putative testis-specific GATA1 target genes (Ref. 94). Further research has to clarify whether there is a functional redundancy between GATA factors in the testis.

During urogenital development, *GATA4* is expressed in somatic ovarian and testicular cell lineages, and is suggested to have an important regulatory role in gonadal gene expression (Fig. 2) (Ref. 95). Mouse embryos conditionally deficient in *GATA4* show no formation of the genital ridge, the structure which differentiates into either testis or ovary (Ref. 96). *GATA4^ki/ki^* mice and *FOG2^−/−^* mice display defects in the gonadogenesis in both sexes (Ref. 97). SRY (Y chromosome-linked testis-determining gene), MIS (Mullerian inhibiting substance) and SOX9 expression, which is critical for testis formation, are dependent on GATA4 × FOG2 interaction (Ref. 98). Recently, a signalling cascade was suggested describing transduction of the p38 mitogen-activated protein kinase (MAPK) pathway by MAP3K4 and GADD45G which leads to GATA4 phosphorylation and thereby activation. Phosphorylated GATA4 then binds and activates the *SRY* promoter (Ref. 99).

The *GATA4* gene has also been implicated in a disorder of sex development (DSD). A *GATA4* mutation, which abrogates the binding with FOG2, was discovered in a family with both CHD and 46,XY DSD (Table 3) (Ref. 100). The phenotype closely resembles that of the mouse *GATA4^ki/ki^* model (Ref. 97). The data described above indicate that GATA4, in combination with FOG2, is necessary for proper mammalian sex differentiation.

Murine GATA5 is expressed in the urogenital ridge during foetal development (Ref. 63). *GATA5*^−/−^ female mice exhibit abnormalities of the genitourinary tract including malpositioning of the urogenital sinus, vagina and urethra, whereas males are unaffected (Table 2). These defects suggest that early morphogenic movements in the lower genitourinary tract are disrupted in the absence of GATA5. GATA5 and GATA6 are coexpressed in the developing urogenital ridge but do not seem to have entirely overlapping functions during development of the female genitourinary system (Ref. 9).

GATA6 is expressed during both testicular and ovarian fetal development (Fig. 2) (Ref. 63). In the developing gonads, GATA4 and GATA6 have overlapping, but distinct expression patterns, which suggest different roles for these transcription factors. In addition, it is also possible that these factors complement each other's functions because GATA4 and GATA6 are expressed in similar cell types in the testis and ovary (Refs 101, 102).

Loss of GATA6 expression has been found in ovarian cancer and has been associated with hypoacetylation of histones H3 and H4 and loss of H3K4me3 at the promoter region (Ref. 90). Downregulation of GATA6 expression results in nuclear deformation and aneuploidy of ovarian surface epithelial cells (Ref. 103). In contrast to other cancers, these data indicate a tumour suppressor role for GATA6 in ovarian cancer. Tumour suppressing activities are also suggested for GATA4 and GATA5 whereas introduction of these genes into ovarian tumour cell lines greatly inhibits cell growth and survival (Ref. 104).

During pronephros formation human GATA3 expression is already detected in the nephric duct (Fig. 2) (Ref. 105). Subsequently, ureter tips and the collecting duct system of the metanephros are formed, which both show GATA3 expression (Ref. 106). Inactivation of the murine *GATA3* locus results in a morphologically abnormal nephric duct with an aberrant elongation path, loss of ureteric bud and a severe growth disturbance of de mesonephros due to the disturbance of a regulatory cascade consisting of GATA3 with β-catenin as upstream regulator and *Ret* as downstream target (Ref. 107).

In humans, *GATA3* haploinsufficiency leads to the HDR syndrome, a rare and complex disease characterised by the combination of HDR, associated with *GATA3* mutations (Table 3, Supplemental Table 1) (Ref. 108). The majority of these mutations leads to loss of DNA binding caused by a disrupted ZnF2, or altered FOG2 interaction and/or DNA binding affinity by a disrupted ZnF1 (Table 3). Most of the HDR probands without *GATA3* mutations do not have renal abnormalities and no *GATA3* mutations are found in patients with isolated hypoparathyreoidism (Ref. 109). This suggests that *GATA3* mutations are highly penetrant and result in the HDR phenotype. In addition, *GATA3^+/−^* mice show small size parathyroids resulting in failure to correct hypocalcaemia similar to HDR patients (Ref. 110). When *GATA3* is specifically deleted in the developing inner ear, defective formation of the cochlear prosensory domain and loss of spiral ganglion neurons is shown (Ref. 111). However, the exact mechanisms leading to the HDR phenotype remain to be elucidated.

### Respiratory tract

The mammalian lung develops from budding of the foregut endoderm, in which both GATA4 and GATA6 are expressed. In vitro analysis of lung development from *GATA4^ki/ki^* mice show abnormal lobar development, revealing *GATA4* as a candidate for FOG2-mediated early pulmonary development (Ref. 112). GATA6-regulated Wnt signalling controls the balance between bronchioalveolar stem cell expansion and epithelial differentiation required for both lung development and regeneration after lung injury (Ref. 113).

However, data about defects in GATA factors in lung diseases are scarce. Recently, *GATA2* requirement for oncogenic *Kras*-driven lung tumorigenenis was reported. Moreover, inhibition of GATA2 regulated pathways in mice with *KRAS* mutant non-small cell lung cancer results in tumour regression (Ref. 114). Finally, a lung cancer susceptibility locus downstream of *GATA3* was identified (Ref. 115).

### Mammary gland

Using GATA3/LacZ knock-in mice, GATA3 expression is observed at the earliest stages of embryonic mammary development (Fig. 2). During puberty GATA3 is expressed in the terminal-end buds and within the adult mammary gland only in luminal epithelial cells. Targeted *GATA3* deletion at different stages of the embryonic mammary development showed loss or absence of mammary primordia and nipples (Ref. 116). Postnatal *GATA3* deletion resulted in loss of mammary gland development, and diminished expression of luminal differentiation markers, which indicates an important role of *GATA3* in the luminal epithelium (Refs 116, 117). Loss of the o*estrogen receptor α* (*ERα*) expression is observed in both *GATA3* knock-out mice and *FOG-2* knock-out mice (Ref. 117). Involvement of GATA3 and ERα in a positive cross-regulatory loop, which has been shown in breast cancer, may be an explanation for these phenomena (Ref. 118). Collectively, these data show that GATA3 is essential during embryonic development as well as the postnatal occurring morphogenesis (Ref. 116). Furthermore, GATA3 directs luminal differentiation of progenitor cells and is needed for active maintenance of the differentiated luminal phenotype (Ref. 117).

The crucial role of GATA3 in the mammary gland is further demonstrated by the observation of *GATA3* mutations in ~10% of human breast cancers. The spectrum of somatic mutations is diverse and cluster predominantly in the vicinity of the highly conserved C-terminal second zinc-finger (Table 3; Supplemental Table 1) (Ref. 119). Restoration of *GATA3* in breast cancer cell lines leads to differentiation, suppressed tumour dissemination (Ref. 120), slower growth rates and induction of genes involved in luminal cell differentiation (Ref. 121). Thereby, GATA3 expression leads to reduced breast tumour outgrowth and inhibits pulmonary metastasis due to repression of metastasis-associated genes (Ref. 122). Recently it was described that GATA3 induces miR-29b expression, which in turn represses metastasis by changing tumour microenvironment (Ref. 123). Together these data indicate that *GATA3* might function as a tumour suppressor gene. In vitro- and in vivo data support this potential tumour suppressor function because loss of GATA3 leads to tumor progression and tumour dissemination in a murine luminal breast cancer model (Ref. 120). Prognostic and predictive features of GATA3 as a biomarker in breast cancer are discussed below in the clinical applications section.

### Central Nervous System (CNS)

GATA2 is expressed early during CNS development in murine embryos (Fig. 2) (Ref. 124). Despite early lethality of *GATA2*^−/−^ embryos (Table 2), several studies show that GATA2 is required for the development of sympathetic neurons (Ref. 125), serotonergic hindbrain neurons (Ref. 126), GABAergic midbrain neurons (Ref. 127), retinorecipient neurons (Ref. 128) and for the generation and cell fate determination of V2b spinal interneurons (Ref. 129). *GATA2^−/−^* embryos lack both GATA2 and GATA3 expression in the CNS, which indicates dependence of GATA3 expression on functional GATA2 during early differentiation of the neural tube (Ref. 130). The expression pattern of *GATA3* during brain development is very similar to GATA2. *GATA3^−/−^* murine embryos also die early during embryonic development (Table 2) and have severe abnormalities of the brain and spinal cord (Ref. 7). Loss of *GATA3* results in reduced Th (tyrosine hydroxylase) and Dbh (dopamine β-hydroxylase) transcripts, which consequently leads to noradrenaline deficiency in the SNS. Administration of catecholamine intermediates to pregnant female *GATA3^+/−^* mice rescues *GATA3^−/−^* murine embryos, thereby partially unraveling the *GATA3* loss-induced lethality (Ref. 131). A transcriptional network, which includes GATA3 (Ref. 132), is essential for cell survival and differentiation of sympathetic neurons during embryonic development as well as during adult life (Ref. 133).

GATA4 is expressed in the embryonic and adult CNS and acts as a negative regulator of astrocyte proliferation and growth (Fig. 2) (Ref. 134). In the adult mouse and human, *GATA6* is expressed in neurons, astrocytes, choroids plexus epithelium and endothelial cells (Fig. 2) (Ref. 135).

Loss of expression of GATA4 and GATA6 occurs in glioblastoma multiforme (GBM). Both *GATA4/6* gene promoters were found to be methylated and for *GATA4* also somatic mutations were found (Refs 136, 137). Limited evidence indicates that GATA4 regulates apoptosis-related genes in cultured GBM cell lines (Ref. 136). *GATA6* was identified in a mouse astrocytoma model as a novel tumour suppressor gene. Knockdown of GATA6 expression in RasV12 or *p53^−/−^* astrocytes led to acceleration of tumourigenesis. Mutations of *GATA6* occur during malignant progression of murine and human astrocytomas (Ref. 135).

## Regulation of GATA genes and proteins in disease

Although mainly *GATA* gene mutations have been described above, chromosomal alterations as well as regulation of *GATA* genes and proteins on transcriptional and post-transcriptional levels can also contribute to disease development.

Recently it has been shown that combined tet methylcytosine dioxygenase 2 (*TET2*) and fms related tyrosine kinase 3 (*FLT3*) mutations regulate epigenetic silencing of *GATA2* by promotor hypermethylation in human AML (Ref. 138). In clear cell renal cell carcinomas downregulation of GATA3 expression by promoter hypermethylation results in decreased expression of TbetaRIII, a protein with tumour suppressor features, during disease progression (Ref. 139). Presence of suppressive histone (H3K27) trimethylation of *GATA3* together with absence of the GATA3 protein in anaplastic large cell lymphoma implicates epigenetical contribution in the pathogenesis of this disease (Ref. 140). Clues about the transcriptional regulation of the *GATA4* and *GATA6* genes come from a SUMO-specific protease 2 (SENP2) knockout model. These mice have reduced expression of GATA4 and GATA6 and defects in the embryonic heart. In SENP2 deficient embryos sumoylation of CBX4, accumulates and occupies the promoters of *GATA4* and *GATA6*, thereby leading to transcriptional repression (Ref. 141).

*GATA4* is located at chromosome 8p, a chromosomal locus frequently deleted in multiple tumour types such as colorectal and oesophageal cancer (Refs 142, 143). Alternatively *GATA4* can be downregulated via epigenetic silencing, such as hypoacetylation of histones H3 and H4 (Ref. 90) and promoter CpG island hypermethylation, which has been observed in colorectal, gastric, oesophageal, lung, ovarian and HPV-driven oropharyngeal cancer, in GBM and in diffuse large B-cell lymphoma (Refs 80, 78, 88, 89, 104, 136, 144, 145). In contrast, *GATA4* amplification is recently described in certain gastric cancer which indicates a more oncogenic function (Ref. 92). Further studies are needed to unravel the molecular mechanisms of *GATA4* amplified in comparison with *GATA4* methylated gastric cancers.

*GATA5* is located at chromosome 20q13, a locus which is often amplified and methylated in multiple cancer types. No coding sequence mutations in *GATA4* and *GATA5* have been described so far in colorectal- and breast cancer (Refs 146, 147). However, promoter methylation of *GATA5* might be established in order to downregulate increased gene expression imposed by amplification. Identified post-transcriptional modifications on GATA proteins include acetylation, phosphorylation and methylation (Fig. 1). Protein stability of GATA2 and GATA3 is regulated by phosphorylation and ubiquitilation. Phosphorylation of GATA3 by respectively Cyclin-dependent kinase 1 (CDK1) and CDK2 was required for F-box/WD repeat-containing protein 7 (Fbw)-7 mediated ubiquitilation and degradation and contributed to precise differentiation of HSCs and T-cell lineages (Refs 148, 149). How GATA acetylation influences transcriptional processes has been investigated for GATA1. It turns out that bromodomain protein Brd3 binds to acetylated GATA1 to regulate the chromatin occupancy at erythroid target genes (Ref. 150). For GATA4 post-transciptional modifications have mainly been studied in the context of hypertrophy of the heart. Activation of GATA4 occurs in part through acetylation by the transcriptional coactivator p300. Takaya et al. identified 4 GATA4 lysine residues that, when mutated, lacked p300-induced acetylation, DNA binding and transcriptional activities (Fig. 1) (Ref. 151). Phosphorylation of p300 by Cdk9 increases the ability of p300 to induce acetylation and DNA binding of GATA4 (Ref. 152). Alternatively, phosphorylation of GATA4 on serine 105 is critical for a productive cardiac hypertrophic response to stress stimulation in adult mice (Ref. 153). Deacetylation of GATA4, and subsequent suppression of transcriptional activation, is mediated by histone deacetylase 2 (HDAC2) and the small homeodomain factor Hopx (Ref. 154). Recently it was reported that the GATA4 protein is methylated by Polycomb-repressive complex 2 member Ezh2. This reduced the interaction with and acetylation by p300, thereby reducing GATA4's transcriptional activity (Ref 155). Together, this emphasises how important post-transciptional modifications are for the regulation of GATA activity.

### Clinical applications of GATA transcription factor alterations

The above mentioned alterations in GATA factors might be applicable as biomarkers for early detection, diagnosis and prediction of prognosis and response to therapy.

#### Early detection markers

Non-invasive early diagnosis of CRC reduces mortality of this disease (Ref. 156). We have shown that *GATA4* promoter methylation is highly prevalent in CRC, suggesting that methylation is an early event in colorectal carcinogenesis. *GATA4* methylation, detected in faecal DNA has potential to be used as a biomarker for improving pre-selection tests for colonoscopy (Ref. 80), especially if the clinical and analytical sensitivity and specificity can be improved by adding additional biomarkers and by introducing sensitive analysis techniques such as for example methylation on beads technology (Ref. 157).

#### Diagnostic markers

The expression of several GATA factors can be helpful in establishing a correct diagnosis. In ovarian cancer loss of GATA4 precedes loss of GATA6 expression and can differentiate between histological subtypes. Loss of both GATA4 and GATA6 expression is found in serous, clear cell and endometrioid ovarian cancer, but their expression can be detected in mucinous carcinomas (Ref. 158).

#### Prognostic markers

As already described above, *GATA1* mutations are found in nearly all AMKL patients with Down syndrome and are already detectable in the precursor lesion TMD. In addition, Down syndrome-neonates without *GATA1* mutations do not develop AMKL (Refs 159, 160). Together, the presence of *GATA1* mutations in Down syndrome-children might be a potential prognostic marker for identifying infants at higher risk of developing AMKL (Ref. 161). Besides having a clinical value in AMKL, prognostic properties of GATA transcription factors are also described in T-ALL. Inherited genetic *GATA3* variants are identified in Philadelphia-like ALL (an ALL subtype with a poor prognosis) and are associated with early treatment response and a higher risk of relapse (Ref. 162).

*GATA3* downregulation has been observed in ER-negative breast cancers and has been described as a strong prognostic indicator of breast cancer. Low GATA3 expression was strongly associated with aggressive disease and poor survival (Ref. 117). Vice versa, breast cancers expressing GATA3- and estrogen regulated genes exhibit a good prognosis and have better relapse-free and overall survival (Ref. 163). GATA3 has been considered to be a better prognostic marker for disease-free survival than commonly used variables such as ER status (Ref. 164) although conflicting data have been published. However, GATA3 expression is highly correlated with the luminal A subtype which has a relatively favourable outcome compared with luminal B and basal-like subtypes (Ref. 165). An explanation could be the downregulation of *p18^INK4C^* transcription by GATA3 resulting in expansion of luminal progenitor cells thereby favouring the development of luminal type breast cancer (Ref. 166).

Recent studies indicate that *GATA2* may be a useful biomarker for predicting prognosis in AML. *GATA2* mutations are frequent in patients with a biallelic *CEBPA* mutation and are associated with a better survival (Ref. 167).

In oropharyngeal carcinomas, a methylation signature of 5 gene promoters, including *GATA4*, correlates with improved survival (Ref. 144). Eventually, loss of expression of GATA4 in GBM is associated with unfavourable patient survival (Ref. 136).

Recently it has been described that low GATA6 expression in lung adenocarcinomas is linked to increased incidence of metastasis and poor outcome (Ref. 168).

#### Predictive markers

Whole genome sequencing of samples from patients with ER-positive breast cancer, participating in aromatase inhibitor clinical trials identified 18 significantly mutated genes, including *GATA3*. Mutant *GATA3* correlated with suppression of proliferation upon aromatase inhibitor treatment and might therefore be a positive predictive marker for aromatase inhibitor response (Ref. 169).

Re-expression of GATA4 in GBM cells conferred sensitivity to temozolomide, a DNA alkylating agent used in GBM therapy (Ref. 136).

Recently, *GATA5* methylation was described as a potential predictive marker for patients with high-risk non-muscle-invasive bladder tumours. These patients had a better survival after treatment with Bacillus Calmette-Guérin (BCG) when *GATA5* was methylated (Ref. 170).

#### Therapeutic interventions

For regenerative medicine the generation of functional differentiated cell types is of great therapeutic interest. Since heart disease occurs frequently and the heart has little regenerative capacity after damage, procedures are sought that can transdifferentiate fibroblast into cardiac myocytes. A cocktail of transcription factors, including GATA4 converts cardiac non-myocytes into cardiomyocyte-like cells in vivo, and alleviates cardiac injury (Refs 171, 172). Also in mouse liver engineering experiments GATA4 was one of the essential factors that contributed to the conversion of fibroblasts into functional hepatocyte-like cells (Ref. 173). These induced cells were able to restore liver function in half of fumarylacetoacetate-hydrolase-deficient mice. *GATA4* is thus one of the pivotal genes that in combination with other transcription factors can be utilised to improve heart and liver function after damage. These promising results are the first steps for bringing regenerative medicine to the clinic. More knowledge of the different GATA protein functions and their downstream target genes is necessary before therapeutic strategies can be developed.

## Conclusions and future perspectives

An increasing number of studies are being published, describing expression and function of *GATA* genes during development in different species.

Causal relationships between aberrations in *GATA* genes and several human diseases have become apparent. Numerous mutations in the *GATA* genes have been described above. Many disease-associated mutations are located in and around the Zinc finger regions. As those mutations are not specifically limited to the two Zinc fingers themselves, it is clear that the whole region is important for the proteins to be fully operational. Most likely mutations hinder the correct folding of the proteins and thereby obstruct GATA proteins from binding to their relevant binding partners. The application of next-generation sequencing technologies through whole-genome, whole-exome and whole-transcriptome approaches allows for substantial advances, which is expected to reveal more disease-associated alterations whithin *GATA* genes.

A better understanding of the regulation of GATA factors on transcriptional, translational and post-translational levels will give more leads to how GATAs can be used as biomarkers. Prospective clinical trials, based on these data, are necessary to determine the translational value of *GATA* genes as biomarkers.

## Supplementary material

For supplementary material accompanying this paper visit http://dx.doi.org/10.1017/erm.2016.2. click here to view supplementary material
