# Supplementary material for: The emerging role of GATA transcription factors in development and disease
Source: Expert Rev Mol Med. 2016 Mar 8;18:e3. doi: 10.1017/erm.2016.2 (PMC4836206; doi:10.1017/erm.2016.2)
Supplement: Supplementary file 1 [file S1462399416000028sup001.docx]

**Supplementary Table 1: Disease related mutations of GATA1 – GATA6 zinc fingers with clinical phenotype from previously reported studies as depicted in figure 1.**

| **Gene** | **Amino acid change** | **Phenotype** | **Literature reference** |
| --- | --- | --- | --- |
| GATA1 | Val205Met | XLT | (Ref. [1](#_ENREF_1)) |
| GATA1 | Gly208Arg | XLT, XLT with MDS | (Ref. [2](#_ENREF_2), [3](#_ENREF_3), [4](#_ENREF_4)) |
| GATA1 | Gly208Ser | XLT | (Ref. [5](#_ENREF_5)) |
| GATA1 | Arg216Gln | XLTT | (Ref. [6](#_ENREF_6), [7](#_ENREF_7), [8](#_ENREF_8), [9](#_ENREF_9)) |
| GATA1 | Arg216Trp | CEP | (Ref. [10](#_ENREF_10)) |
| GATA1 | Asp218Gly | XLT | (Ref. [11](#_ENREF_11)) |
| GATA1 | Asp218Tyr | XLT | (Ref. [12](#_ENREF_12)) |
| GATA2 | Arg293Gln | AML | (Ref. [13](#_ENREF_13)) |
| GATA2 | Arg307Leu | AML | (Ref. [14](#_ENREF_14)) |
| GATA2 | Arg307Trp | AML | (Ref. [15](#_ENREF_15)) |
| GATA2 | Arg308Pro | AML | (Ref. [14](#_ENREF_14), [15](#_ENREF_15), [16](#_ENREF_16)) |
| GATA2 | Asn317His | AML | (Ref. [13](#_ENREF_13)) |
| GATA2 | Asn317Ile | AML | (Ref. [14](#_ENREF_14), [15](#_ENREF_15)) |
| GATA2 | Asn317Ser | AML | (Ref. [14](#_ENREF_14)) |
| GATA2 | Ala318Gly | AML | (Ref. [13](#_ENREF_13)) |
| GATA2 | Ala318fs | MDS with myeloid transformation | (Ref. [17](#_ENREF_17)) |
| GATA2 | Ala318Thr | AML | (Ref. [13](#_ENREF_13), [15](#_ENREF_15)) |
| GATA2 | Ala318Val | (paediatric) AML | (Ref. [13](#_ENREF_13), [14](#_ENREF_14), [15](#_ENREF_15), [18](#_ENREF_18)) |
| GATA2 | Gly320Asp | AML | (Ref. [13](#_ENREF_13), [14](#_ENREF_14)) |
| GATA2 | Gly320Val | AML | (Ref. [15](#_ENREF_15)) |
| GATA2 | Leu321Arg | AML | (Ref. [14](#_ENREF_14)) |
| GATA2 | Leu321His | AML | (Ref. [14](#_ENREF_14)) |
| GATA2 | Leu321Phe | AML | (Ref. [13](#_ENREF_13), [14](#_ENREF_14), [15](#_ENREF_15)) |
| GATA2 | Leu321Pro | AML | (Ref. [13](#_ENREF_13), [15](#_ENREF_15)) |
| GATA2 | Leu321Val | AML | (Ref. [13](#_ENREF_13), [14](#_ENREF_14)) |
| GATA2 | Gln328Pro | AML | (Ref. [13](#_ENREF_13)) |
| GATA2 | Asn329Gln | AML | (Ref. [13](#_ENREF_13), [14](#_ENREF_14), [15](#_ENREF_15)) |
| GATA2 | Arg330Leu | AML | (Ref. [15](#_ENREF_15)) |
| GATA2 | Arg330Pro | AML | (Ref. [15](#_ENREF_15)) |
| GATA2 | Arg330* | MDS with myeloid transformation  AML | (Ref. [17](#_ENREF_17), [19](#_ENREF_19)) |
| GATA2 | Leu332fs | Emberger syndrome | (Ref. [20](#_ENREF_20)) |
| GATA2 | Arg337* | Emberger syndrome | (Ref. [21](#_ENREF_21)) |
| GATA2 | Arg337* | MonoMAC  MDS  Emberger syndrome | (Ref. [17](#_ENREF_17), [21](#_ENREF_21), [22](#_ENREF_22)) |
| GATA2 | Ser340_Asn381del | MDS  MonoMAC | (Ref. [23](#_ENREF_23)) |
| GATA2 | Ala341fs | Emberger syndrome | (Ref. [21](#_ENREF_21), [23](#_ENREF_23)) |
| GATA2 | Ala342fs | NK-cell deficiency | (Ref. [24](#_ENREF_24)) |
| GATA2 | Ala342Thr | Paediatric AML | (Ref. [25](#_ENREF_25)) |
| GATA2 | Ala350_Asn351ins8 | AML | (Ref. [16](#_ENREF_16)) |
| GATA2 | Thr354Lys | AML | (Ref. [14](#_ENREF_14)) |
| GATA2 | Thr354Met | MonoMAC  AML  MDS  NK-cell deficiency | (Ref. [17](#_ENREF_17), [20](#_ENREF_20), [22](#_ENREF_22), [23](#_ENREF_23), [24](#_ENREF_24), [26](#_ENREF_26), [27](#_ENREF_27), [28](#_ENREF_28), [29](#_ENREF_29), [30](#_ENREF_30), [31](#_ENREF_31)) |
| GATA2 | Thr355del | Familial MDS | (Ref. [27](#_ENREF_27)) |
| GATA2 | Thr357Ser | Paediatric AML | (Ref. [25](#_ENREF_25)) |
| GATA2 | Thr358Lys | AML/MDS | (Ref. [32](#_ENREF_32)) |
| GATA2 | Leu359Val | AML/MDS  acute myeloid transformation of CML  CML | (Ref. [32](#_ENREF_32), [33](#_ENREF_33), [34](#_ENREF_34)) |
| GATA2 | Arg361Cys | Emberger syndrome  MonoMAC and MDS  MDS with myeloid transformation | (Ref. [17](#_ENREF_17), [22](#_ENREF_22), [23](#_ENREF_23)) |
| GATA2 | Arg361His | AML  MDS with myeloid transformation | (Ref. [14](#_ENREF_14), [17](#_ENREF_17)) |
| GATA2 | Arg361Leu | Emberger syndrome | (Ref. [21](#_ENREF_21)) |
| GATA2 | Arg362_Asn365del | MDS with myeloid transformation  DCML | (Ref. [24](#_ENREF_24), [29](#_ENREF_29), [35](#_ENREF_35)) |
| GATA2 | Arg362Gln | (paediatric) AML | (Ref. [14](#_ENREF_14), [18](#_ENREF_18), [25](#_ENREF_25)) |
| GATA2 | Arg362Gly | Paediatric AML | (Ref. [18](#_ENREF_18), [25](#_ENREF_25)) |
| GATA2 | Arg362Pro | Paediatric AML | (Ref. [18](#_ENREF_18), [25](#_ENREF_25)) |
| GATA2 | Arg362fs | Paediatric AML | (Ref. [18](#_ENREF_18)) |
| GATA2 | Asp367fs | Monocytopenia  MDS with myeloid transformation  MonoMAC | (Ref. [17](#_ENREF_17), [22](#_ENREF_22)) |
| GATA2 | Asn371Lys | MDS with myeloid transformation  DCML | (Ref. [17](#_ENREF_17), [29](#_ENREF_29)) |
| GATA2 | Ala372Thr | AML | (Ref. [19](#_ENREF_19)) |
| GATA2 | Cys373Arg | Emberger syndrome | (Ref. [21](#_ENREF_21)) |
| GATA2 | Cys373_Tyr377del | MDS with myeloid transformation | (Ref. [17](#_ENREF_17)) |
| GATA2 | Leu375Ile | Paediatric AML | (Ref. [25](#_ENREF_25)) |
| GATA2 | Leu379Gln | AML | (Ref. [14](#_ENREF_14)) |
| GATA2 | Met388Thr | MonoMAC  Monocytopenia  Laryngeal cancer | (Ref. [17](#_ENREF_17), [22](#_ENREF_22)) |
| GATA2 | Met388Val | DCML | (Ref. [19](#_ENREF_19)) |
| GATA2 | Lys390del | MDS | (Ref. [23](#_ENREF_23)) |
| GATA2 | Arg396Gln | MonoMAC  DCML and MDS  MDS with myeloid transformation  AML  Low B-cell | (Ref. [17](#_ENREF_17), [19](#_ENREF_19), [22](#_ENREF_22), [31](#_ENREF_31), [36](#_ENREF_36)) |
| GATA2 | Arg396Glu | MDS  AML | (Ref. [26](#_ENREF_26)) |
| GATA2 | Arg396Trp | MonoMAC and MDS  MDS with myeloid transformation | (Ref. [17](#_ENREF_17)) |
| GATA2 | Arg398Gln | MonoMAC | (Ref. [23](#_ENREF_23)) |
| GATA2 | Arg398Trp | DCML  MonoMAC  MDS (with myeloid transformation)  CMML  NK-cell deficiency | (Ref. [17](#_ENREF_17), [22](#_ENREF_22), [23](#_ENREF_23), [24](#_ENREF_24), [31](#_ENREF_31), [37](#_ENREF_37)) |
| GATA3 | Arg261Gly | HDR syndrome | (Ref. [38](#_ENREF_38)) |
| GATA3 | Ser270fs | T-ALL | (Ref. [39](#_ENREF_39)) |
| GATA3 | Thr271Ile | HDR syndrome | (Ref. [40](#_ENREF_40), [41](#_ENREF_41)) |
| GATA3 | Trp274Arg | HDR syndrome | (Ref. [42](#_ENREF_42)) |
| GATA3 | Trp274Leu | HDR syndrome | (Ref. [43](#_ENREF_43)) |
| GATA3 | Arg275Gln | T-ALL | (Ref. [39](#_ENREF_39)) |
| GATA3 | Arg275Trp | T-ALL | (Ref. [39](#_ENREF_39)) |
| GATA3 | Arg276Pro | HDR syndrome | (Ref. [44](#_ENREF_44)) |
| GATA3 | Arg276* | HDR syndrome | (Ref. [45](#_ENREF_45), [46](#_ENREF_46)) |
| GATA3 | Asn285Thr | T-ALL | (Ref. [39](#_ENREF_39)) |
| GATA3 | Met293Lys | Breast cancer | (Ref. [47](#_ENREF_47), [48](#_ENREF_48), [49](#_ENREF_49)) |
| GATA3 | Lys302* | HDR syndrome | (Ref. [38](#_ENREF_38), [50](#_ENREF_50)) |
| GATA3 | Ser308fs | Breast cancer | (Ref. [51](#_ENREF_51)) |
| GATA3 | Ser308* | Breast cancer | (Ref. [51](#_ENREF_51)) |
| GATA3 | Ala309_Ala313del | T-ALL | (Ref. [39](#_ENREF_39)) |
| GATA3 | Thr315_Ala318del | HDR syndrome | (Ref. [45](#_ENREF_45)) |
| GATA3 | Thr315fs | Breast cancer | (Ref. [51](#_ENREF_51)) |
| GATA3 | Cys317fs | HDR syndrome | (Ref. [52](#_ENREF_52)) |
| GATA3 | Cys317Arg | HDR syndrome | (Ref. [53](#_ENREF_53)) |
| GATA3 | Cys317Ser | HDR syndrome | (Ref. [38](#_ENREF_38)) |
| GATA3 | Asn319Lys | HDR syndrome | (Ref. [53](#_ENREF_53)) |
| GATA3 | Cys320Ser | HDR syndrome | (Ref. [54](#_ENREF_54)) |
| GATA3 | Thr322fs | Breast cancer | (Ref. [49](#_ENREF_49)) |
| GATA3 | Arg329fs | Breast cancer | (Ref. [47](#_ENREF_47), [48](#_ENREF_48), [55](#_ENREF_55)) |
| GATA3 | Arg329del | Breast cancer | (Ref. [55](#_ENREF_55)) |
| GATA3 | Arg330Lys | Breast cancer | (Ref. [47](#_ENREF_47), [48](#_ENREF_48)) |
| GATA3 | Asn331fs | Breast cancer | (Ref. [47](#_ENREF_47), [48](#_ENREF_48)) |
| GATA3 | Ala332fs | Breast cancer | (Ref. [47](#_ENREF_47), [48](#_ENREF_48), [56](#_ENREF_56)) |
| GATA3 | Asp335fs | Breast cancer | (Ref. [56](#_ENREF_56)) |
| GATA3 | Cys341Tyr | HDR syndrome | (Ref. [57](#_ENREF_57)) |
| GATA3 | Leu343Phe | Breast cancer | (Ref. [51](#_ENREF_51)) |
| GATA3 | Tyr345fs | Breast cancer | (Ref. [49](#_ENREF_49)) |
| GATA3 | Leu347Val | T-ALL | (Ref. [39](#_ENREF_39)) |
| GATA3 | Leu347Arg | HDR syndrome | (Ref. [46](#_ENREF_46)) |
| GATA3 | Arg352Ser | HDR syndrome | (Ref. [58](#_ENREF_58)) |
| GATA3 | Leu354fs | Breast cancer | (Ref. [49](#_ENREF_49)) |
| GATA3 | Leu354* | HDR syndrome | (Ref. [38](#_ENREF_38), [46](#_ENREF_46)) |
| GATA3 | Met356* | HDR syndrome | (Ref. [42](#_ENREF_42), [46](#_ENREF_46)) |
| GATA3 | Met356fs | Breast cancer | (Ref. [49](#_ENREF_49)) |
| GATA3 | Lys357* | Breast cancer | (Ref. [49](#_ENREF_49)) |
| GATA3 | Glu359fs | Breast cancer | (Ref. [49](#_ENREF_49)) |
| GATA3 | Arg364Gly | Breast cancer | (Ref. [49](#_ENREF_49)) |
| GATA3 | Arg364Ser | Breast cancer | (Ref. [47](#_ENREF_47), [48](#_ENREF_48)) |
| GATA3 | Arg366Leu | Breast cancer | (Ref. [51](#_ENREF_51)) |
| GATA3 | Arg366* | HDR syndrome  Breast cancer | (Ref. [42](#_ENREF_42), [48](#_ENREF_48), [51](#_ENREF_51), [53](#_ENREF_53), [59](#_ENREF_59)) |
| GATA3 | Ser369* | HDR syndrome | (Ref. [53](#_ENREF_53)) |
| GATA4 | Glu216Asp | CHD (TOF) | (Ref. [60](#_ENREF_60)) |
| GATA4 | Gly221Arg | Anomalies of human testicular development | (Ref. [61](#_ENREF_61)) |
| GATA4 | Met223Thr | CHD (VSD) | (Ref. [62](#_ENREF_62)) |
| GATA4 | Pro226= | CHD (VSD) | (Ref. [63](#_ENREF_63)) |
| GATA4 | Pro226fs | CHD (AVSD) | (Ref. [62](#_ENREF_62)) |
| GATA4 | Pro226Gln | CHD (DCM) | (Ref. [64](#_ENREF_64)) |
| GATA4 | Arg229Ser | CHD (VSD / ASD / AVSD) | (Ref. [62](#_ENREF_62)) |
| GATA4 | Thr233= | CHD (DORV / PFO / TOF / PA / VSD / AS) | (Ref. [65](#_ENREF_65), [66](#_ENREF_66), [67](#_ENREF_67), [68](#_ENREF_68)) |
| GATA4 | Gly234Ser | CHD (AVSD) | (Ref. [62](#_ENREF_62)) |
| GATA4 | Asn239= | CHD (VSD) | (Ref. [62](#_ENREF_62)) |
| GATA4 | Asn239Ser | CHD (VSD) | (Ref. [62](#_ENREF_62)) |
| GATA4 | Cys241= | CHD (TGA / VSD / ASD) | (Ref. [63](#_ENREF_63), [66](#_ENREF_66)) |
| GATA4 | Tyr244Cys | CHD (VSD / AVSD) | (Ref. [62](#_ENREF_62)) |
| GATA4 | Tyr244= | CHD (AVSD / TOF /PA / LSVC) | (Ref. [68](#_ENREF_68)) |
| GATA4 | Met247Thr | CHD (AF) | (Ref. [69](#_ENREF_69)) |
| GATA4 | Asn248= | CHD (ASD / VSD) | (Ref. [63](#_ENREF_63)) |
| GATA4 | Asn248Ser | CHD (ASD / AVSD) | (Ref. [62](#_ENREF_62)) |
| GATA4 | Ile250Asn | CHD (VSD) | (Ref. [63](#_ENREF_63)) |
| GATA4 | Arg252Pro | CHD (AVSD) | (Ref. [62](#_ENREF_62)) |
| GATA4 | Ile255Thr | CHD (ASD) | (Ref. [62](#_ENREF_62)) |
| GATA4 | Arg260Gln | CHD (VSD) | (Ref. [62](#_ENREF_62)) |
| GATA4 | Leu261Pro | CHD (VSD / ASD) | (Ref. [62](#_ENREF_62)) |
| GATA4 | Ser262= | CHD (TOF / PA) | (Ref. [68](#_ENREF_68)) |
| GATA4 | Ala263Gly | CHD (VSD) | (Ref. [70](#_ENREF_70)) |
| GATA4 | Arg266* | CHD (AVSD) | (Ref. [62](#_ENREF_62)) |
| GATA4 | Val267Met | CHD (ASD / PDA) | (Ref. [71](#_ENREF_71), [72](#_ENREF_72)) |
| GATA4 | Cys271Ser | CHD (DCM) | (Ref. [73](#_ENREF_73)) |
| GATA4 | Asn273Lys | Pancreatic agenesis | (Ref. [74](#_ENREF_74)) |
| GATA4 | Asn273Ser | CHD (AVSD) | (Ref. [62](#_ENREF_62)) |
| GATA4 | Cys274= | CHD (TOF / PA / DORV / VSD / TGA / ASD) | (Ref. [66](#_ENREF_66), [67](#_ENREF_67)) |
| GATA4 | Thr277Ile | CHD (AVSD) | (Ref. [62](#_ENREF_62)) |
| GATA4 | Thr279Ser | CHD (DCM) | (Ref. [64](#_ENREF_64)) |
| GATA4 | Thr280Met | CHD (ASD) | (Ref. [75](#_ENREF_75)) |
| GATA4 | Arg283His | CHD (AVSD) | (Ref. [62](#_ENREF_62)) |
| GATA4 | Asn285Ser | CHD (TOF) | (Ref. [76](#_ENREF_76)) |
| GATA4 | Asn285Lys | CHD (AVSD) | (Ref. [62](#_ENREF_62)) |
| GATA4 | Val291Leu | CHD (DCM) | (Ref. [77](#_ENREF_77)) |
| GATA4 | Cys292Arg | CHD (VSD / ASD / AVSD) | (Ref. [62](#_ENREF_62), [78](#_ENREF_78)) |
| GATA4 | Ala294Val | CHD (ASD) | (Ref. [62](#_ENREF_62)) |
| GATA4 | Gly296Arg | CHD (VSD) | (Ref. [79](#_ENREF_79)) |
| GATA4 | Gly296Cys | CHD (ASD / PS) | (Ref. [80](#_ENREF_80)) |
| GATA4 | Gly296Ser | CHD (ASD / VSD / AVSD / PS) | (Ref. [81](#_ENREF_81), [82](#_ENREF_82)) |
| GATA4 | His302Arg | CHD (AVSD) | (Ref. [62](#_ENREF_62)) |
| GATA4 | Met310Val | CHD (ASD) | (Ref. [83](#_ENREF_83)) |
| GATA4 | Gln316Glu | CHD (ASD) | (Ref. [67](#_ENREF_67)) |
| GATA4 | Arg318Trp | CHD (ASD)  Pancreatic agenesis | (Ref. [84](#_ENREF_84)) |
| GATA4 | Lys319Glu | CHD (ASD / PS) | (Ref. [85](#_ENREF_85)) |
| GATA4 | Leu325= | CHD (ASD) | (Ref. [63](#_ENREF_63)) |
| GATA4 | Lys329Asn | CHD | (Ref. [86](#_ENREF_86)) |
| GATA5 | Arg187Gly | CHD (TOF) | (Ref. [87](#_ENREF_87)) |
| GATA5 | Val190Ala | CHD (VSD / TOF) | (Ref. [88](#_ENREF_88)) |
| GATA5 | Leu199Val | CHD (VSD) | (Ref. [89](#_ENREF_89)) |
| GATA5 | Trp200Gly | CHD (AF) | (Ref. [90](#_ENREF_90)) |
| GATA5 | Asp203Glu | CHD (TOF) | (Ref. [91](#_ENREF_91)) |
| GATA5 | Asp203= | CHD (BAV) | (Ref. [92](#_ENREF_92)) |
| GATA5 | His207Arg | CHD (TOF) | (Ref. [87](#_ENREF_87)) |
| GATA5 | Tyr208* | CHD (TOF) | (Ref. [91](#_ENREF_91)) |
| GATA5 | Cys210Gly | CHD (AF) | (Ref. [93](#_ENREF_93)) |
| GATA5 | Lys218Thr | CHD (AF) | (Ref. [94](#_ENREF_94)) |
| GATA5 | Leu226= | CHD (BAV) | (Ref. [92](#_ENREF_92)) |
| GATA5 | Leu233Pro | CHD (BAV) | (Ref. [95](#_ENREF_95), [96](#_ENREF_96)) |
| GATA5 | Thr252Pro | CHD (BAV) | (Ref. [97](#_ENREF_97)) |
| GATA5 | Ala266Pro | CHD (AF / TOF) | (Ref. [88](#_ENREF_88), [94](#_ENREF_94)) |
| GATA5 | His274Arg | CHD (VSD) | (Ref. [88](#_ENREF_88)) |
| GATA5 | Lys284= | CHD (BAV) | (Ref. [92](#_ENREF_92)) |
| GATA6 | Gly394Cys | CHD (TOF) | (Ref. [98](#_ENREF_98)) |
| GATA6 | Asp404Tyr | CHD (TOF) | (Ref. [99](#_ENREF_99)) |
| GATA6 | Cys447Arg | Diabetes | (Ref. [100](#_ENREF_100)) |
| GATA6 | Thr452Ala | Pancreatic agenesis  CHD (ASD) | (Ref. [101](#_ENREF_101)) |
| GATA6 | Arg456Cys | Pancreatic agenesis  CHD (VSD / PTA / TOF)  ConHD | (Ref. [101](#_ENREF_101), [102](#_ENREF_102)) |
| GATA6 | Arg456His | Pancreatic agenesis  CHD (e.g. PTA and VSD) | (Ref. [101](#_ENREF_101)) |
| GATA6 | Glu460* | CHD (TOF) | (Ref. [99](#_ENREF_99)) |
| GATA6 | Asn466Asp | Pancreatic agenesis  PTA | (Ref. [101](#_ENREF_101)) |
| GATA6 | Asn466His | CHD (PTA) | (Ref. [103](#_ENREF_103)) |
| GATA6 | Asn466Ser | CHD  Permanent neonatal diabetes | (Ref. [104](#_ENREF_104)) |
| GATA6 | Ala467Thr | Pancreatic agenesis  CHD (ASD / PS) | (Ref. [101](#_ENREF_101)) |
| GATA6 | Gly469Glu | Pancreatic agenesis | (Ref. [100](#_ENREF_100)) |
| GATA6 | Gly469Val | CHD (AF) | (Ref. [105](#_ENREF_105)) |
| GATA6 | Lys473Gln | Pancreatic agenesis  CHD (ASD) | (Ref. [101](#_ENREF_101)) |
| GATA6 | Arg479Gly | Pancreatic agenesis  CHD (e.g. AVSD, PS) | (Ref. [100](#_ENREF_100)) |
| GATA6 | Met483fs | Pancreatic agenesis  CHD | (Ref. [101](#_ENREF_101)) |
| GATA6 | Arg493* | Pancreatic agenesis  CHD | (Ref. [106](#_ENREF_106)) |
| GATA6 | Lys500fs | Pancreatic agenesis  CHD (e.g. VSD and PS) | (Ref. [101](#_ENREF_101)) |
| GATA6 | Lys502fs | Pancreatic agenesis  CHD (VSD / PTA) | (Ref. [107](#_ENREF_107)) |

*AF* atrial fibrillation; *AML* acute myeloid leukemia; *AS* aortic stenosis; *ASD* atrial septal defect; *AVSD* atrial ventricular septal defect; *BAV* bicuspid aortic valve; *CEP* congenital erythropoietic porphyria; *CHD* congenital heart disease; *CML* chronic myeloid leukemia; *CMML* chronic myelomonocytic leukemia; *ConHD* congenital hernia diaphragmatica; *DCM* dilated cardiomyopathy; *DCML* dendritic cell, monocyte, B lymphocyte, and natural killer lymphocyte deficiency; *DORV* double-outlet right ventricle; *HDR* syndrome hypoparathyroidism, sensorineural deafness and renal insufficiency; *MDS* myelodysplastic syndrome; *PA* pulmonary atresia; *PDA* patent ductus arteriosis; *PFO* patent foramen ovale; *PS* pulmonary valve stenosis; *T-ALL* T-cell acute lymphoblastic leukemia; *TGA* transposition of the great arteries; *TOF* Tetralogy of Fallot; *VSD* ventricular septal defect; *XLTT* X-linked thrombocytopenia with thalassemia; *XLT* X-linked thrombocytopenia

**REFERENCES**

1. Nichols, K.E., et al. (2000) Familial dyserythropoietic anaemia and thrombocytopenia due to an inherited mutation in GATA1. Nature genetics **24**(3), 266-70

2. Duhrsen, U., et al. (2011) Long-term outcome of hemizygous and heterozygous carriers of a germline GATA1 (G208R) mutation. Annals of hematology **90**(3), 301-6

3. Kratz, C.P., et al. (2008) Congenital transfusion-dependent anemia and thrombocytopenia with myelodysplasia due to a recurrent GATA1(G208R) germline mutation. Leukemia **22**(2), 432-4

4. Del Vecchio, G.C., et al. (2005) Dyserythropoietic anemia and thrombocytopenia due to a novel mutation in GATA-1. Acta Haematol **114**(2), 113-6

5. Mehaffey, M.G., et al. (2001) X-linked thrombocytopenia caused by a novel mutation of GATA-1. Blood **98**(9), 2681-8

6. Yu, C., et al. (2002) X-linked thrombocytopenia with thalassemia from a mutation in the amino finger of GATA-1 affecting DNA binding rather than FOG-1 interaction. Blood **100**(6), 2040-5

7. Tubman, V.N., et al. (2007) X-linked gray platelet syndrome due to a GATA1 Arg216Gln mutation. Blood **109**(8), 3297-9

8. Balduini, C.L., et al. (2004) Effects of the R216Q mutation of GATA-1 on erythropoiesis and megakaryocytopoiesis. Thromb Haemost **91**(1), 129-40

9. Hughan, S.C., et al. (2005) Selective impairment of platelet activation to collagen in the absence of GATA1. Blood **105**(11), 4369-76

10. Phillips, J.D., et al. (2007) Congenital erythropoietic porphyria due to a mutation in GATA1: the first trans-acting mutation causative for a human porphyria. Blood **109**(6), 2618-21

11. Freson, K., et al. (2001) Platelet characteristics in patients with X-linked macrothrombocytopenia because of a novel GATA1 mutation. Blood **98**(1), 85-92

12. Freson, K., et al. (2002) Different substitutions at residue D218 of the X-linked transcription factor GATA1 lead to altered clinical severity of macrothrombocytopenia and anemia and are associated with variable skewed X inactivation. Human molecular genetics **11**(2), 147-52

13. Greif, P.A., et al. (2012) GATA2 zinc finger 1 mutations associated with biallelic CEBPA mutations define a unique genetic entity of acute myeloid leukemia. Blood **120**(2), 395-403

14. Fasan, A., et al. (2013) GATA2 mutations are frequent in intermediate-risk karyotype AML with biallelic CEBPA mutations and are associated with favorable prognosis. Leukemia **27**(2), 482-5

15. Green, C.L., et al. (2013) GATA2 mutations in sporadic and familial acute myeloid leukaemia patients with CEBPA mutations. British journal of haematology **161**(5), 701-5

16. Niimi, K., et al. (2013) GATA2 zinc finger 2 mutation found in acute myeloid leukemia impairs myeloid differentiation. Leukemia research reports **2**(1), 21-5

17. West, R.R., et al. (2014) Acquired ASXL1 mutations are common in patients with inherited GATA2 mutations and correlate with myeloid transformation. Haematologica **99**(2), 276-81

18. Luesink, M., et al. (2012) High GATA2 expression is a poor prognostic marker in pediatric acute myeloid leukemia. Blood **120**(10), 2064-75

19. Pasquet, M., et al. (2013) High frequency of GATA2 mutations in patients with mild chronic neutropenia evolving to MonoMac syndrome, myelodysplasia, and acute myeloid leukemia. Blood **121**(5), 822-9

20. Kazenwadel, J., et al. (2012) Loss-of-function germline GATA2 mutations in patients with MDS/AML or MonoMAC syndrome and primary lymphedema reveal a key role for GATA2 in the lymphatic vasculature. Blood **119**(5), 1283-91

21. Ostergaard, P., et al. (2011) Mutations in GATA2 cause primary lymphedema associated with a predisposition to acute myeloid leukemia (Emberger syndrome). Nature genetics **43**(10), 929-31

22. Hsu, A.P., et al. (2013) GATA2 haploinsufficiency caused by mutations in a conserved intronic element leads to MonoMAC syndrome. Blood **121**(19), 3830-7, S1-7

23. Dickinson, R.E., et al. (2014) The evolution of cellular deficiency in GATA2 mutation. Blood **123**(6), 863-74

24. Mace, E.M., et al. (2013) Mutations in GATA2 cause human NK cell deficiency with specific loss of the CD56(bright) subset. Blood **121**(14), 2669-77

25. Shiba, N., et al. (2014) Mutations of the GATA2 and CEBPA genes in paediatric acute myeloid leukaemia. British journal of haematology **164**(1), 142-5

26. Holme, H., et al. (2012) Marked genetic heterogeneity in familial myelodysplasia/acute myeloid leukaemia. British journal of haematology **158**(2), 242-8

27. Hahn, C.N., et al. (2011) Heritable GATA2 mutations associated with familial myelodysplastic syndrome and acute myeloid leukemia. Nature genetics **43**(10), 1012-7

28. Bodor, C., et al. (2012) Germ-line GATA2 p.THR354MET mutation in familial myelodysplastic syndrome with acquired monosomy 7 and ASXL1 mutation demonstrating rapid onset and poor survival. Haematologica **97**(6), 890-4

29. Hsu, A.P., et al. (2011) Mutations in GATA2 are associated with the autosomal dominant and sporadic monocytopenia and mycobacterial infection (MonoMAC) syndrome. Blood **118**(10), 2653-5

30. Bigley, V., et al. (2011) The human syndrome of dendritic cell, monocyte, B and NK lymphoid deficiency. The Journal of experimental medicine **208**(2), 227-34

31. Cuellar-Rodriguez, J., et al. (2011) Successful allogeneic hematopoietic stem cell transplantation for GATA2 deficiency. Blood **118**(13), 3715-20

32. Gao, J., et al. (2014) Heritable GATA2 mutations associated with familial AML-MDS: a case report and review of literature. Journal of hematology & oncology **7**(1), 36

33. Zhang, S.J., et al. (2008) Gain-of-function mutation of GATA-2 in acute myeloid transformation of chronic myeloid leukemia. Proceedings of the National Academy of Sciences of the United States of America **105**(6), 2076-81

34. Zhang, S.J., Shi, J.Y. and Li, J.Y. (2009) GATA-2 L359 V mutation is exclusively associated with CML progression but not other hematological malignancies and GATA-2 P250A is a novel single nucleotide polymorphism. Leukemia research **33**(8), 1141-3

35. West, E.S., et al. (2014) Generalized verrucosis in a patient with GATA2 deficiency. The British journal of dermatology **170**(5), 1182-6

36. Ishida, H., et al. (2012) GATA-2 anomaly and clinical phenotype of a sporadic case of lymphedema, dendritic cell, monocyte, B- and NK-cell (DCML) deficiency, and myelodysplasia. European journal of pediatrics **171**(8), 1273-6

37. Dickinson, R.E., et al. (2011) Exome sequencing identifies GATA-2 mutation as the cause of dendritic cell, monocyte, B and NK lymphoid deficiency. Blood **118**(10), 2656-8

38. Nakamura, A., et al. (2011) Molecular analysis of the GATA3 gene in five Japanese patients with HDR syndrome. Endocrine journal **58**(2), 123-30

39. Zhang, J., et al. (2012) The genetic basis of early T-cell precursor acute lymphoblastic leukaemia. Nature **481**(7380), 157-63

40. Gomes, T.S., et al. (2012) HDR syndrome: a follow-up genotype-phenotype analysis of a de novo missense Thr272Ile mutation in exon 4 of GATA3. Klinische Padiatrie **224**(7), 452-4

41. Gaynor, K.U., et al. (2009) A missense GATA3 mutation, Thr272Ile, causes the hypoparathyroidism, deafness, and renal dysplasia syndrome. The Journal of clinical endocrinology and metabolism **94**(10), 3897-904

42. Muroya, K., et al. (2001) GATA3 abnormalities and the phenotypic spectrum of HDR syndrome. Journal of medical genetics **38**(6), 374-80

43. Fukami, M., et al. (2011) GATA3 abnormalities in six patients with HDR syndrome. Endocrine journal **58**(2), 117-21

44. Zahirieh, A., et al. (2005) Functional analysis of a novel GATA3 mutation in a family with the hypoparathyroidism, deafness, and renal dysplasia syndrome. The Journal of clinical endocrinology and metabolism **90**(4), 2445-50

45. Van Esch, H., et al. (2000) GATA3 haplo-insufficiency causes human HDR syndrome. Nature **406**(6794), 419-22

46. Ali, A., et al. (2007) Functional characterization of GATA3 mutations causing the hypoparathyroidism-deafness-renal (HDR) dysplasia syndrome: insight into mechanisms of DNA binding by the GATA3 transcription factor. Human molecular genetics **16**(3), 265-75

47. (2012) Comprehensive molecular portraits of human breast tumours. Nature **490**(7418), 61-70

48. Jiang, Y.Z., et al. (2014) GATA3 mutations define a unique subtype of luminal-like breast cancer with improved survival. Cancer **120**(9), 1329-37

49. Ellis, M.J., et al. (2012) Whole-genome analysis informs breast cancer response to aromatase inhibition. Nature **486**(7403), 353-60

50. Mino, Y., et al. (2005) Identification of a novel insertion mutation in GATA3 with HDR syndrome. Clinical and experimental nephrology **9**(1), 58-61

51. Usary, J., et al. (2004) Mutation of GATA3 in human breast tumors. Oncogene **23**(46), 7669-78

52. Chenouard, A., et al. (2013) Renal phenotypic variability in HDR syndrome: glomerular nephropathy as a novel finding. European journal of pediatrics **172**(1), 107-10

53. Nesbit, M.A., et al. (2004) Characterization of GATA3 mutations in the hypoparathyroidism, deafness, and renal dysplasia (HDR) syndrome. The Journal of biological chemistry **279**(21), 22624-34

54. Ohta, M., et al. (2011) Novel dominant-negative mutant of GATA3 in HDR syndrome. Journal of molecular medicine **89**(1), 43-50

55. Gaynor, K.U., et al. (2013) GATA3 mutations found in breast cancers may be associated with aberrant nuclear localization, reduced transactivation and cell invasiveness. Hormones & cancer **4**(3), 123-39

56. Wheler, J.J., et al. (2014) Unique molecular signatures as a hallmark of patients with metastatic breast cancer: implications for current treatment paradigms. Oncotarget **5**(9), 2349-54

57. Moldovan, O., et al. (2011) A new case of HDR syndrome with severe female genital tract malformation: comment on "Novel mutation in the gene encoding the GATA3 transcription factor in a Spanish familial case of hypoparathyroidism, deafness, and renal dysplasia (HDR) syndrome with female genital tract malformations" by Hernandez et al. American journal of medical genetics. Part A **155A**(9), 2329-30

58. Chiu, W.Y., et al. (2006) Identification of three novel mutations in the GATA3 gene responsible for familial hypoparathyroidism and deafness in the Chinese population. The Journal of clinical endocrinology and metabolism **91**(11), 4587-92

59. Sun, Y., et al. (2009) Germinal mosaicism of GATA3 in a family with HDR syndrome. American journal of medical genetics. Part A **149A**(4), 776-8

60. Nemer, G., et al. (2006) A novel mutation in the GATA4 gene in patients with Tetralogy of Fallot. Human mutation **27**(3), 293-4

61. Lourenco, D., et al. (2011) Loss-of-function mutation in GATA4 causes anomalies of human testicular development. Proceedings of the National Academy of Sciences of the United States of America **108**(4), 1597-602

62. Reamon-Buettner, S.M. and Borlak, J. (2005) GATA4 zinc finger mutations as a molecular rationale for septation defects of the human heart. Journal of medical genetics **42**(5), e32

63. Wang, E., et al. (2013) Identification of functional mutations in GATA4 in patients with congenital heart disease. PloS one **8**(4), e62138

64. Li, J., et al. (2014) Prevalence and spectrum of GATA4 mutations associated with sporadic dilated cardiomyopathy. Gene **548**(2), 174-81

65. Reamon-Buettner, S.M., Cho, S.H. and Borlak, J. (2007) Mutations in the 3'-untranslated region of GATA4 as molecular hotspots for congenital heart disease (CHD). BMC medical genetics **8**, 38

66. Butler, T.L., et al. (2010) GATA4 mutations in 357 unrelated patients with congenital heart malformation. Genetic testing and molecular biomarkers **14**(6), 797-802

67. Tomita-Mitchell, A., et al. (2007) GATA4 sequence variants in patients with congenital heart disease. Journal of medical genetics **44**(12), 779-83

68. Schluterman, M.K., et al. (2007) Screening and biochemical analysis of GATA4 sequence variations identified in patients with congenital heart disease. American journal of medical genetics. Part A **143A**(8), 817-23

69. Posch, M.G., et al. (2010) Mutations in the cardiac transcription factor GATA4 in patients with lone atrial fibrillation. European journal of medical genetics **53**(4), 201-3

70. Xiong, F., et al. (2013) Analyses of GATA4, NKX2.5, and TFAP2B genes in subjects from southern China with sporadic congenital heart disease. Cardiovascular pathology : the official journal of the Society for Cardiovascular Pathology **22**(2), 141-5

71. Wang, J., et al. (2010) [Genetic screening for novel GATA4 mutations associated with congenital atrial septal defect]. Zhonghua xin xue guan bing za zhi **38**(5), 429-34

72. Tang, Z.H., et al. (2006) Two novel missense mutations of GATA4 gene in Chinese patients with sporadic congenital heart defects. Zhonghua yi xue yi chuan xue za zhi = Zhonghua yixue yichuanxue zazhi = Chinese journal of medical genetics **23**(2), 134-7

73. Li, R.G., et al. (2013) GATA4 loss-of-function mutation underlies familial dilated cardiomyopathy. Biochemical and biophysical research communications **439**(4), 591-6

74. Shaw-Smith, C., et al. (2014) GATA4 mutations are a cause of neonatal and childhood-onset diabetes. Diabetes **63**(8), 2888-94

75. Chen, Y., et al. (2010) A novel mutation of GATA4 in a familial atrial septal defect. Clinica chimica acta; international journal of clinical chemistry **411**(21-22), 1741-5

76. Yang, Y.Q., et al. (2013) GATA4 loss-of-function mutations underlie familial tetralogy of fallot. Human mutation **34**(12), 1662-71

77. Zhao, L., et al. (2014) A novel GATA4 loss-of-function mutation responsible for familial dilated cardiomyopathy. International journal of molecular medicine **33**(3), 654-60

78. Reamon-Buettner, S.M., Spanel-Borowski, K. and Borlak, J. (2006) Bridging the gap between anatomy and molecular genetics for an improved understanding of congenital heart disease. Annals of anatomy = Anatomischer Anzeiger : official organ of the Anatomische Gesellschaft **188**(3), 213-20

79. Wang, J., et al. (2011) A novel GATA4 mutation responsible for congenital ventricular septal defects. International journal of molecular medicine **28**(4), 557-64

80. Rajagopal, S.K., et al. (2007) Spectrum of heart disease associated with murine and human GATA4 mutation. J Mol Cell Cardiol **43**(6), 677-85

81. Garg, V., et al. (2003) GATA4 mutations cause human congenital heart defects and reveal an interaction with TBX5. Nature **424**(6947), 443-7

82. Sarkozy, A., et al. (2005) Spectrum of atrial septal defects associated with mutations of NKX2.5 and GATA4 transcription factors. Journal of medical genetics **42**(2), e16

83. Chen, Y., et al. (2010) A novel mutation in GATA4 gene associated with dominant inherited familial atrial septal defect. The Journal of thoracic and cardiovascular surgery **140**(3), 684-7

84. D'Amato, E., et al. (2010) Genetic investigation in an Italian child with an unusual association of atrial septal defect, attributable to a new familial GATA4 gene mutation, and neonatal diabetes due to pancreatic agenesis. Diabetic medicine : a journal of the British Diabetic Association **27**(10), 1195-200

85. Xiang, R., et al. (2013) A novel mutation of GATA4 (K319E) is responsible for familial atrial septal defect and pulmonary valve stenosis. Gene

86. Wang, J., et al. (2010) [Novel GATA4 mutations identified in patients with congenital heart disease]. Zhonghua yi xue za zhi **90**(10), 667-71

87. Wei, D., et al. (2013) GATA5 loss-of-function mutations underlie tetralogy of fallot. International journal of medical sciences **10**(1), 34-42

88. Jiang, J.Q., et al. (2013) Prevalence and spectrum of GATA5 mutations associated with congenital heart disease. International journal of cardiology **165**(3), 570-3

89. Wei, D., et al. (2013) GATA5 loss-of-function mutation responsible for the congenital ventriculoseptal defect. Pediatric cardiology **34**(3), 504-11

90. Wang, X.H., et al. (2013) A novel GATA5 loss-of-function mutation underlies lone atrial fibrillation. International journal of molecular medicine **31**(1), 43-50

91. Huang, R.T., et al. (2014) Somatic GATA5 mutations in sporadic tetralogy of Fallot. International journal of molecular medicine **33**(5), 1227-35

92. Foffa, I., et al. (2013) Sequencing of NOTCH1, GATA5, TGFBR1 and TGFBR2 genes in familial cases of bicuspid aortic valve. BMC medical genetics **14**, 44

93. Gu, J.Y., et al. (2012) Novel GATA5 loss-of-function mutations underlie familial atrial fibrillation. Clinics **67**(12), 1393-9

94. Yang, Y.Q., et al. (2012) Mutational spectrum of the GATA5 gene associated with familial atrial fibrillation. International journal of cardiology **157**(2), 305-7

95. Francis, C., et al. (2014) 95 Identification Of Likely Pathogenic Variants In Patients With Bicuspid Aortic Valve: Correlation Of Complex Genotype With A More Severe Aortic Phenotype. Heart **100**(Suppl 3), A55-A56

96. Bonachea, E.M., et al. (2014) Rare GATA5 sequence variants identified in individuals with bicuspid aortic valve. Pediatric research **76**(2), 211-6

97. Shi, L.M., et al. (2014) GATA5 loss-of-function mutations associated with congenital bicuspid aortic valve. International journal of molecular medicine **33**(5), 1219-26

98. Huang, R.T., et al. (2013) Somatic mutations in the GATA6 gene underlie sporadic tetralogy of Fallot. International journal of molecular medicine **31**(1), 51-8

99. Wang, J., et al. (2012) Novel GATA6 mutations associated with congenital ventricular septal defect or tetralogy of fallot. DNA and cell biology **31**(11), 1610-7

100. De Franco, E., et al. (2013) GATA6 mutations cause a broad phenotypic spectrum of diabetes from pancreatic agenesis to adult-onset diabetes without exocrine insufficiency. Diabetes **62**(3), 993-7

101. Allen, H.L., et al. (2012) GATA6 haploinsufficiency causes pancreatic agenesis in humans. Nature genetics **44**(1), 20-22

102. Yu, L., et al. (2014) Whole exome sequencing identifies de novo mutations in GATA6 associated with congenital diaphragmatic hernia. Journal of medical genetics **51**(3), 197-202

103. Kodo, K., et al. (2009) GATA6 mutations cause human cardiac outflow tract defects by disrupting semaphorin-plexin signaling. Proceedings of the National Academy of Sciences of the United States of America **106**(33), 13933-8

104. Catli, G., et al. (2013) A novel GATA6 mutation leading to congenital heart defects and permanent neonatal diabetes: a case report. Diabetes & metabolism **39**(4), 370-4

105. Li, J., et al. (2012) Novel GATA6 loss-of-function mutation responsible for familial atrial fibrillation. International journal of molecular medicine **30**(4), 783-90

106. Suzuki, S., et al. (2014) A case of pancreatic agenesis and congenital heart defects with a novel GATA6 nonsense mutation: evidence of haploinsufficiency due to nonsense-mediated mRNA decay. American journal of medical genetics. Part A **164A**(2), 476-9

107. Bonnefond, A., et al. (2012) GATA6 inactivating mutations are associated with heart defects and, inconsistently, with pancreatic agenesis and diabetes. Diabetologia **55**(10), 2845-7
